# Spectroscopic observation of oxygen dissociation on nitrogen-doped graphene

**DOI:** 10.1038/s41598-017-08651-1

**Published:** 2017-08-11

**Authors:** Mattia Scardamaglia, Toma Susi, Claudia Struzzi, Rony Snyders, Giovanni Di Santo, Luca Petaccia, Carla Bittencourt

**Affiliations:** 1 Chemistry of Interaction Plasma Surface (ChIPS), University of Mons, 7000 Mons, Belgium; 2 University of Vienna, Faculty of Physics, Boltzmanngasse 5, A-1090 Vienna, Austria; 3 Elettra Sincrotrone Trieste, Strada Statale 14 km 163.5, 34149 Trieste, Italy

## Abstract

Carbon nanomaterials’ reactivity towards oxygen is very poor, limiting their potential applications. However, nitrogen doping is an established way to introduce active sites that facilitate interaction with gases. This boosts the materials’ reactivity for bio-/gas sensing and enhances their catalytic performance for the oxygen reduction reaction. Despite this interest, the role of differently bonded nitrogen dopants in the interaction with oxygen is obscured by experimental challenges and has so far resisted clear conclusions. We study the interaction of molecular oxygen with graphene doped via nitrogen plasma by *in situ* high-resolution synchrotron techniques, supported by density functional theory core level simulations. The interaction leads to oxygen dissociation and the formation of carbon-oxygen single bonds on graphene, along with a band gap opening and a rounding of the Dirac cone. The change of the N 1 *s* core level signal indicates that graphitic nitrogen is involved in the observed mechanism: the adsorbed oxygen molecule is dissociated and the two O atoms chemisorb with epoxy bonds to the nearest carbon neighbours of the graphitic nitrogen. Our findings help resolve existing controversies and offer compelling new evidence of the ORR pathway.

## Introduction

Due to their surprisingly high electrocatalytic activity, nitrogen-doped carbon nanomaterials have been proposed as low-cost candidates to substitute platinum catalysts for the oxygen reduction reaction (ORR)^1–4^ in fuel cells cathodes. While pristine graphene and carbon nanotubes (CNTs) are chemically inert, the active sites induced by functionalization with nitrogen lower the energy barrier for O_2_ dissociation^5–7^, the first step of the ORR. This makes doping appealing also for applications other than heterogeneous catalysis, such as chemical or bio-sensing where the possibility to detect ppm levels of gases such as NO_2_, CO, H_2_ has been demonstrated, thus possibly avoiding the use of expensive metals^8, 9^. The activationz barrier for oxygen dissociation on pristine graphene is higher than on CNTs (2.71 eV and 1.61 eV, respectively)^5^ since the CNT curvature has a strong effect in lowering the required energy^6^.

A nitrogen atom can be incorporated into the hexagonal carbon network in three main configurations: N simply substituting a single C atom is called *graphitic*, a substitution with a vacancy as a neighbour is called *pyridinic* (both with *sp²* hybridization), or the N can be included in a pentagonal ring (either with *sp³* or *sp²* hybridization) or in a local lattice distortion (such as Stone-Thrower-Wales defects)^10^ to form a *pyrrolic* configuration. Theoretical models show different energy barriers for oxygen molecule adsorption and dissociation depending on the nitrogen configuration^5^. Since their five valence electrons are distributed differently, graphitic and pyridinic nitrogen atoms have different effects on the electronic properties of graphene: the graphitic nitrogen contributes with three σ bonds and two *p*_*z*_ orbitals, with the extra electron available for conduction in a partially-occupied π* band; on the contrary, the pyridinic nitrogen only forms two σ bonds with the neighbouring carbon atoms, one electron occupies the *p*_*z*_ orbital, and the other two form an electron lone pair. Since there is no occupation of the π* band in the pyridinic nitrogen, it does not behave as an electron dopant like graphitic N does (nor is it thought to be responsible for good ORR activity^5–7^). Furthermore, due to the proximity of the carbon vacancy, the pyridinic-vacancy complex has a hole doping effect. These predictions were recently experimentally confirmed by following the Dirac cone shift upon modification of the graphitic/pyridinic ratio by thermal treatments of graphene^11^ and by the intercalation of different metal atoms^12^.

From an experimental point of view, the role of these active sites in the ORR is still not well understood and the mechanism for the enhanced catalytic activity is controversial^13–16^. Three main issues make direct experimental observation very challenging: (i) low doping level, (ii) poor control over the bonding of the nitrogen dopants and (iii) the indirect nature of experimental studies reported so far, which often involve steps where the contamination of the sample cannot be controlled. To overcome limitations caused by commonly used N-doped graphene synthesis methods (i.e. the introduction of dopant species to the feedstock during chemical vapour deposition (CVD), or via wet chemistry), we used a post-synthesis plasma treatment^11, 17^. This method is clean, it does not add undesired oxygen contamination, and it does not destroy the *sp²* character of the graphene network even while reaching a high concentration of N (on the order of 10 at.%)^17, 18^. Further temperature treatments allow both the passivation of carbon defects and the conversion of pyridinic-N into graphitic-N, resulting in a larger density of states at the Fermi level with increasing graphitic-N level^11, 19^. With our work, we build further steps to link the field of catalysis with fundamental surface science, making substantial progress in the comprehension of the role of differently bonded nitrogen dopants in the interaction with molecular oxygen.

Using advanced synchrotron techniques, we investigated the response of nitrogen functionalities to the interaction with O_2_. The graphene synthesis and measurements were performed *in situ* in an ultra-high-vacuum (UHV) environment and the interaction with molecular oxygen was followed by the direct measurement of the core levels, valence band and unoccupied states of the system with synchrotron-based photoemission (XPS: X-ray photoelectron spectroscopy, and ARPES: angle-resolved photoemission spectroscopy) and absorption (NEXAFS: near-edge absorption fine structure) techniques. The electronic structure of N-graphene was probed in a contamination-free environment before and after UHV exposure to oxygen. The experimental measurements are interpreted in the light of core level binding energy shifts calculated via density functional theory (DFT), leading to a detailed atomistic picture of the oxygen dissociation process.

## Results and Discussion

**Figure 1:**
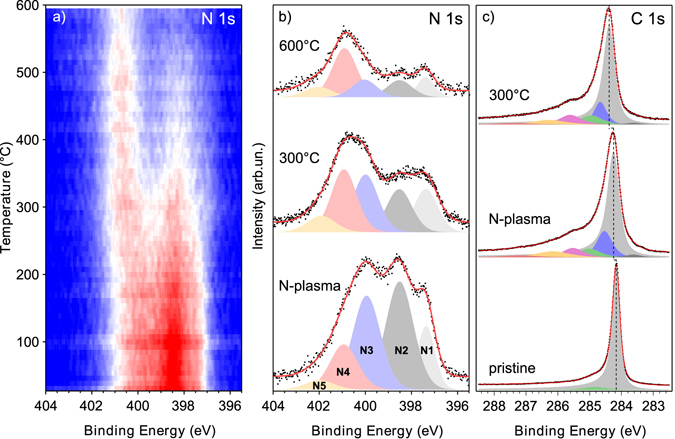
(**a**) Temperature-programmed XPS data as a 2D trace of the spectral intensity as a function of BE and temperature (higher to lower intensity represented by false colours from red to blue). (**b**) N 1 *s* spectra recorded at room temperature after plasma exposure and temperature ramps respectively up to 300 and 600 °C. The two spectra after annealing come from two distinct temperature ramps on the two samples. (**c**) C 1 *s* core level spectra of pristine graphene/Ir(111), after nitrogen plasma exposure, and after annealing at 300 °C. Black dots are the experimental data, the red continuous line is the fitting curve and the black dashed line is a guide for the eye. The pristine graphene C 1 *s* spectrum is multiplied by 0.5 for ease comparison.

We prepared two graphene/Ir(111) samples that we exposed for 15 minutes to the downstream of a plasma source fed with N_2_ gas. As evaluated by the survey XPS spectra, nitrogen concentrations of 7.3 and 7.8 at.% are reached in the two samples. The thermal stability and rearrangement of the nitrogen species were studied by temperature-programmed XPS: the two identically prepared samples were annealed with a controlled temperature ramp of 0.4 °C/s respectively up to 300 and 600 °C, while following the N 1 *s* core level signal evolution. Figure 1a shows two-dimensional intensity-projected plots for the 600 °C case. The high-resolution N 1 *s* core level spectra, recorded at room temperature, for the as-doped and after each annealing treatment are shown in Fig. 1b. The three main nitrogen components are straightforward to individuate by XPS since they appear at different binding energies: pyridinic at 398.5 eV (N2), pyrrolic at 400.0 eV (N3) and graphitic at 400.9 eV (N4), in agreement with literature^18, 20^ and with our previous findings on carbon nanomaterials doped via nitrogen plasma^17, 19^. At the binding energy corresponding to pyrrolic nitrogen, many other defective components may be found, such as C-N single bonds, three-coordinated N atom in a Stone-Wales (SW) defects or nitrogen adatoms^18, 21^. A fourth component at a higher binding energy (≈ 402.0 eV, N5) has been recently also assigned to graphitic nitrogen, with the N atom thought to occupy a substitutional position close to a crystallite edge^22^. Overlapping at a similar binding energy, nitrogen oxide could also contribute, which we can exclude here since no trace of oxygen was detected after the plasma exposure. A further component at a lower binding energy (397.4 eV, N1) is due to nitrogen atoms in the carbon network closer to the metallic substrate^18^, likely due to the pinning of graphene^23^. The 300 °C treatment slightly reduces the amount of nitrogen to 5.7 at.%. The main effect of annealing is the rising intensity of the high-binding energy side in the N 1 *s* spectra, corresponding to the graphitic components that pass from 1.5 to 2.1 at.% in absolute content at the expense of pyridinic and pyrrolic components, which decrease from 6.4 to 3.6 at.%. Judging from the slight loss in N content, these less stable functionalities partially desorb, and partially convert into graphitic-N. This is further evidenced after reaching 600 °C on the second sample, where the greatest amount (56.3%) of nitrogen present is in graphitic form. The overall nitrogen content decreased to 2.5 at.%, but the amount of graphitic is almost the same as for previous annealing, meaning that the loss is mainly due to pyridinic and pyrrolic functionalities, further confirming the high temperature stability of graphitic nitrogen^17, 18^. The results of the fitting procedure (BE, relative area and total amount) are summarized in Table 1 for both samples.

**Table 1:** Summary of the peak fitting analysis of the N 1 *s* core level spectra of the two samples annealed at 300 and 600 °C and after O_2_ exposure.

	N type (BE in eV)	N1 (397.4)	N2 (398.5)	N3 (400.0)	N4 (400.9)	N5 (402.0)	N6 (399.7)
Sample #1	Rel. A %	13.5	36.4	31.6	15.2	3.3	
[N] = 7.8 at.%	At. %	1.1	2.8	2.5	1.2	0.3	
#1, 300°C	Rel. A %	18.1	19.4	26.0	28.3	8.2	
[N] = 5.7 at.%	At. %	1.0	1.1	1.5	1.6	0.5	
#1, O2 exp	Rel. A %	23.0	23.8	5.1	23.6	7.6	16.9
[N] = 5.1 at.%	At. %	1.2	1.2	0.3	1.2	0.4	0.9
Sample #2	Rel. A %	10.8	44.9	28.1	12.5	3.7	
[N] = 7.3 at.%	At. %	0.8	3.3	2.1	0.9	0.3	
#2, 600°C	Rel. A %	12.1	14.9	16.6	45.1	11.2	
[N] = 2.5 at.%	At. %	0.3	0.4	0.4	1.1	0.3	
#2, O2 exp	Rel. A %	17.4	22.2	2.8	32.8	8.4	16.4
[N] = 2.5 at.%	At. %	0.4	0.6	0.1	0.8	0.2	0.4

### Carbon 1 ***s*** levels

Annealing favours the passivation of defects and dangling bonds on the graphene surface, with the filling of carbon vacancies next to the N sites explaining the transformation of pyridinic into graphitic bonding^11, 17^. The C 1 *s* core level of graphene/Ir(111) is shown in Fig. 1c. The main component can be fitted, in agreement with literature^24^, by a sharp asymmetric peak centred at 284.16 eV corresponding to C-C sp², plus a small shoulder at + 0.66 eV that can be attributed to defective carbon (possibly due to the slightly lower growth temperature of graphene/Ir films compared to Ir single crystals). Upon nitrogen plasma exposure, new components appear in the C 1 *s* spectrum at lower binding energies with respect to the main peak (283.34 eV and 283.80 eV), attributed to photoelectrons emitted by carbon atoms near vacancies^25^. A slightly higher binding energy component is likely due to second-nearest carbon neighbours of N atoms (284.53 eV), with additional contributions from *sp²* and *sp³* C-N bonds (285.52 eV, 286.16 eV and 287.21 eV)^18^.

**Figure 2:**
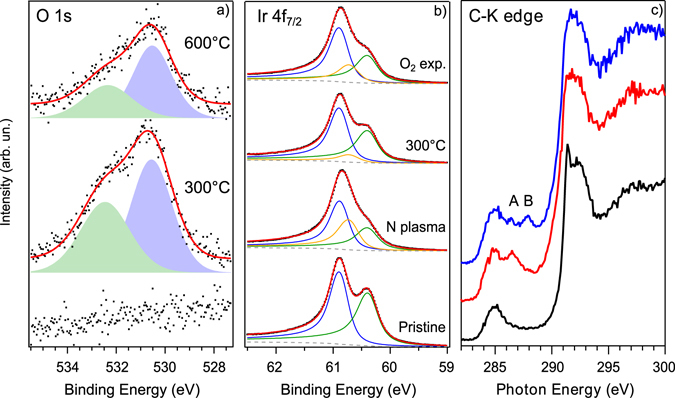
(**a**) O 1 *s* core level spectra before (bottom) and after the exposure to O_2_ for the two samples annealed at 300 and 600 °C after nitrogen plasma; experimental data in black dots, continuous red curve is the fitting result. (**b**) Ir 4*f*_*7/2*_ core level spectra. Ir-bulk component is blue, Ir-surface is green and Ir-surface2 is yellow. A Shirley background (grey dashed line) has been used in the fitting. (**c**) From bottom to top: carbon K-edge NEXAFS spectra of pristine graphene/Ir(111) (black), before (red) and after (blue) exposure to O_2_ for the sample annealed to 300 °C after nitrogen incorporation. The spectra are recorded with linearly polarized light at normal incidence on the sample and with in-plane polarization.

The increase of graphitic nitrogen after annealing at 300 °C is reflected in enhanced *n*-type doping of graphene, deduced by the shift towards higher binding energy of the C-C *sp²* component in the C 1 *s* core level: this peak broadens and shifts with respect to the pristine C 1 *s* position. In particular, a shift of +0.08 eV was evaluated after the plasma exposure and a further shift of +0.22 eV after annealing, in both 300 and 600 °C cases. This behaviour highlights the *n*-doping action of graphitic nitrogen only, counterbalanced by the complex carbon vacancy-pyridinic nitrogen responsible for *p*-doping. In fact, although after annealing the total amount of nitrogen decreases, the shift towards higher binding energy of the C-C *sp²* peak due to *n*-doping increases, thanks to the change in the graphitic/pyridinic ratio^11^. As the annealing has a healing effect on the carbon network with the passivation of vacancies, the two pre-*sp²* peaks are quenched: in particular, the amount of vacancies decreases from 2.3 at.% after the plasma to 1.3 and 0.8 at.% in the two samples respectively annealed at 300 and 600 °C. Such a low amount of residual carbon vacancies testifies the highly crystalline quality of the nitrogen-doped graphene, also in agreement with the observed decrease in pyridinic nitrogen content. The effect of nitrogen implantation in the graphene network is also reflected in the Ir 4*f*_7/2_ core level (see Fig. 2b): among the bulk and surface peaks at 60.89 eV and 60.40 eV, respectively, a new feature appears at 60.72 eV at the expense of the surface component (see Supplementary Table S1 for all the fitting parameters). This new structure is assigned to nitrogen atoms closer to the iridium substrate due to the pinning of graphene^18, 23^, as already verified by the N 1 *s* core level.

After annealing, this component reduces and the original surface peak is restored, in agreement with the observed change in the N 1 *s* line-shape where the graphitic-N, less interacting with iridium, becomes dominant.

### Exposure to oxygen

To study the interaction with molecular oxygen, we exposed both annealed N-graphene/Ir samples *in situ* to 5 × 10^−5^ mbar of O_2_ at a temperature of 200 °C for 30 min, and then recorded XPS and NEXAFS spectra across the C *K*-edge (ARPES is discussed later in this paper). After exposure, there is an oxygen uptake corresponding to 2.0 at.% for the N-graphene annealed up to 300 °C and to 1.2 at.% for the one annealed up to 600 °C. In both cases, two O 1 *s* components are found at 530.6 and 532.3 eV, as shown in Fig. 2a. The temperature we used during oxygen exposure does not allow the etching of graphene, which takes place at higher temperatures even on partially covered iridium^26^. In fact, the intensity of the C 1 *s* does not show any decrease (etching) during the process, indicating the integrity of the graphene flakes, as reported in Supplementary Fig. S1.

Before assigning the O 1 *s* features to a particular bonding, we consider the possibility for molecular oxygen of intercalating between the substrate and the graphene and to dissociate on the metal. Larciprete and co-workers^27^ found a complete intercalation of pristine graphene/Ir(111) for a substrate temperature of 250 °C and a pressure two order of magnitude higher than ours. Even though for defective graphene, this process can be activated at a lower gas dose^27^, we did not see any indication of oxygen intercalation. There are three typical XPS fingerprints of an effective penetration of O_2_ molecules under the graphene layer and their dissociation on the Ir substrate: (1) the appearance of an O 1 *s* peak at 529.8 eV; (2) the transformation of the C 1 *s* which broadens due to the formation of a second component at −0.5 eV by the co-existence of partially intercalated areas until it completely shifts to its new position; (3) the appearance of two new components on the Ir 4*f*_7/2_ core level spectra at the expense of the surface component due to iridium atoms bonding to one (at 60.57 eV) or two (at 61.08 eV) oxygen atoms^28^. The same elements were observed in the experiment of Grånäs and co-workers, where a partial graphene layer on Ir(111) was used^29^. In our case, the C 1 *s* shows a shift of −0.08 eV with respect to the value measured after the annealing of the N-graphene, moving from 284.38 eV to 284.30 eV. This shift is not compatible with oxygen intercalation, since that mechanism does not implicate a gradual progressive shift in the binding energy of the C 1 *s* as in the case of doping. Instead, intercalation causes a peak broadening due to the appearance of a second component arising from O-(2 × 1) intercalated regions^26, 27^. We thus attribute this shift to reduced *n*-doping by the graphitic nitrogen due to absorbed oxygen atoms, as we will show later by core level simulations. Our C 1 *s* lineshape only slightly varies, as reported in Supplementary Fig. S2.

Meanwhile, the Ir 4*f*_7/2_ core level does not show any further peaks indicative of an interaction between the metal and the gas molecules (see Fig. 2b and Supplementary Table S1 for all the fitting parameters): the extra component maintains its position at 60.72 ± 0.02 eV, while a shift of −0.15 eV would be expected for an Ir-O peak. Its limited increase after oxygen interaction can be explained following the work of Vinogradov *et al*.^23^: the oxygen atoms increase the pinning of the carbon layer towards the iridium substrate when adsorbed on the graphene surface. Furthermore, this is also a limiting factor for intercalation, reducing the mobility of O atoms.

Finally, the amount of O in the sample is proportional to the nitrogen initially incorporated; if this were a substrate effect, it would be independent of the doping. As we will show later, the presence of the moiré spots in the LEED pattern after oxygen exposure is a further confirmation of the lack of intercalation that would tend to lift the graphene and make it flat.

### Oxygen 1 ***s*** levels

The above evidence indicates that O_2_ is effectively only interacting with and dissociates on graphene. We ascribe the two components in the O 1 *s* spectrum of Fig. 2a to carbon single-bonded to oxygen in an epoxy (530.6 eV) and ether (532.3 eV) configurations (slightly downshifted from their values in pristine graphene^23, 30^, see below). Epoxy is the most dominant species formed while oxidizing graphene^25, 30, 31^. Ether is usually found at the edges and defects of graphene, and therefore we would expect a decrease of this component when the annealing is performed to a higher temperature (quenching more defects, as also supported by the decreased amount of pyridinic nitrogen). In fact, its relative amount is slightly different in the two samples: ether is 43% for the N-graphene previously annealed to 300 °C, while it is 30% on the one annealed to 600 °C. Together with ethers, some carbonyl groups (C=O) may also form^32^, but their contribution to the O 1 *s* spectrum cannot be disentangled from epoxies because they appear at the same binding energy and their amount is expected to be very low. In fact, DFT calculations^33^ and experimental results^25, 30^ showed that the dominant species is epoxy, followed by ether and finally carbonyl.

### Carbon K-edge

Further information on the oxygen dissociation and the resulting C-O bonding was obtained by absorption spectroscopy at the carbon *K*-edge (Fig. 2c). The spectrum for graphene/Ir(111) is highly dichroic due to the planar symmetry of the system and the intrinsic in-plane (σ*) and out-of-plane (π*) nature of the electronic states^34^. We therefore concentrated on in-plane polarization to suppress the otherwise dominating graphene contribution in the π* region of the spectrum. For clean graphene, we can identify a residual of the C 1 *s* → π* transitions at 285 eV, followed by two peaks at 291.4 and 292.4 eV corresponding to the threshold of transitions to the σ^1^* and σ^2^* orbitals, respectively. Upon nitrogenation and annealing at 300 °C, a new peak appears at 286.4 eV (indicated by the letter A in Fig. 2c), attributed to sp² C-N bonds^35^. After the oxygen exposure, we found a new feature at 287.9 eV (indicated by the letter B in Fig. 2c). This peak was observed after oxidation of graphene/Ir(111) with atomic oxygen by Vinogradov *et al*.^23^ and attributed to ether groups; it was also proposed as an indication of carbon-oxygen single bonds in graphene oxide^36, 37^.

### Core level simulations

All these observations cross-confirm our hypothesis of oxygen dissociation on N-doped graphene resulting in the creation of C-O epoxy and ether bonds, as we have excluded the possibility of O_2_ intercalation beneath graphene and subsequent dissociation catalyzed by the metallic substrate. The role of nitrogen in this mechanism therefore has to be clarified. Theoretical calculations have shown that the redistribution of charge and spin density around nitrogen atoms induces a net positive charge on adjacent carbon atoms, which influences oxygen adsorption^1, 7^. In particular, only three-fold coordinated nitrogen atoms (graphitic and N in a Stone-Wales defect) do actually provide partially occupied π* anti-bonding orbitals, thus acting as active site, contrary to pyridinic-N^5^. In terms of energetics, it is known that O_2_ adsorption on the C-N bridge site is not favourable, and instead takes place on a C-C site close to a N site^6^.

**Figure 3:**
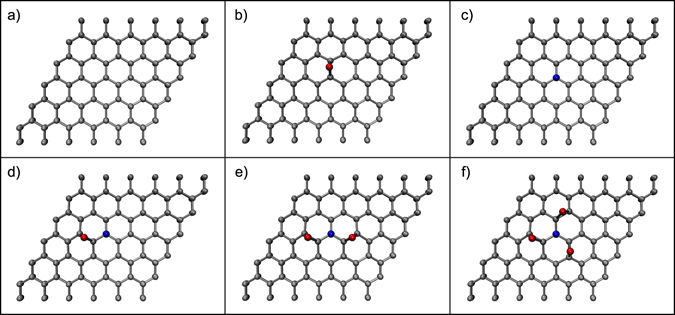
Supercells of DFT-relaxed graphene structures (carbon atoms are coloured grey, oxygen red, and nitrogen blue). (**a**) Pristine and (**b**) epoxide-functionalized graphene; (**c**) graphitic N; (**d**–**f**) graphitic N with (**d**) one, (**e**) two, or (**f**) three epoxide O adsorbed at their preferred binding sites over the N-neighbouring C-C bonds.

To simulate the atomic structures involved and to predict the XPS signatures of differently bonded and oxygenated N sites, we turned to density functional theory (see Methods). While it would be feasible to simulate an entire 524-atom graphene/Ir(111) system to model the C 1 *s* lineshape^38, 39^, as we show in Supplementary Fig. S2, changes in the experimental C 1 *s* line are not very informative. Furthermore, the available computational method is known to give different absolute energies for the core levels of different elements^40^, making it doubtful that such a large and expensive simulation would offer additional insights in the present context. Thus, starting with a relaxed 6 × 6 supercell of graphene in vacuum, we created and relaxed the structure of the graphitic N site, then added between one and three O atoms near their expected absorption sites and relaxed the structures further. For comparison, we also calculated pristine graphene, and added epoxide oxygen. Images of the structures are shown in Fig. 3.

To obtain C, N and O 1 *s* core level binding energies, we calculated total energy differences between the ground state and the first core-excited state, as described in the Methods. There are two factors that complicate a direct interpretation of calculated BE values. One is the system: we simulate a single layer of graphene in vacuum, whereas the experimental sample lies on the Ir(111) substrate. This clearly has some effect on the BEs, since the experimental C 1 *s* is at 284.16 eV instead of the expected value near graphite’s 284.42 eV^41^. However, it is not clear whether all other binding energies are similarly affected, or is the bulk value more sensitive since it is more influenced by the long-range metallic screening that is perturbed by the moiré. The other factor is the known underestimation of the values^42^, which yield a pristine graphene C 1 *s* at 283.827 eV for our simulation parameters, about 0.6 eV (0.02%) lower than the experimental graphite value. For ease of comparison to the experimental data, we have aligned the calculated BEs to the relevant experimental references (pristine graphene C 1 *s* at 284.16 eV, graphitic N 1 *s* at 400.9 eV, pristine graphene epoxide O 1 *s* at 531.2 eV) in Table 2, with the structures referenced to the panels of Fig. 3. Note that such corrections do not affect the chemical shifts of an element in two different local environments, which forms the basis of our conclusions.

**Table 2:** Calculated 1 *s* core level binding energies (BEs). For ease of interpretation, the energies for each element are corrected by a constant factor so that the bolded values (pristine graphene, graphitic N, epoxide O; magnitude of correction denoted below system identifier) match experimental values.

Panel	System (correction in eV)	Aligned 1 *s* BE (eV)							
		C @ N	shift	C @ O	shift	N	shift	O	shift
a	pristine (C 1 *s* + 0.33)	284.16^*^	—	—	—	—	—	—	—
b	pristine >O (O 1 *s* + 0.82)	—	—	285.73	1.57	—	—	**531.20**	**0.00**
c	N_gra_ (N 1 *s* + 0.88)	285.11	0.95	—	—	**400.90**	**0.00**	—	—
d	N_gra_ > O	285.12	0.96	286.73	2.57	400.42	−0.48	530.53	−0.67
e	N_gra_ > O × 2	284.52	0.36	286.62	2.46	399.96	−0.94	530.68	−0.52
f	N_gra_ > O × 3	—	—	286.70	2.54	400.01	−0.89	530.97	−0.23

^*^For pristine graphene, this value refers to the bulk C 1 *s* level.

Upon oxidation, the graphitic N 1 *s* level downshifts by −0.48 eV for a single epoxide O, or −0.94 eV for two epoxide O. The O 1 *s* of epoxide atoms bonded at the N-neighboring C likewise downshift, by −0.67 eV for a single O or by −0.52 eV for two O, in good agreement with our experimental BE that is −0.6 eV downshifted from what is expected on pristine graphene^23, 30^.

### Nitrogen 1 ***s*** levels

**Figure 4:**
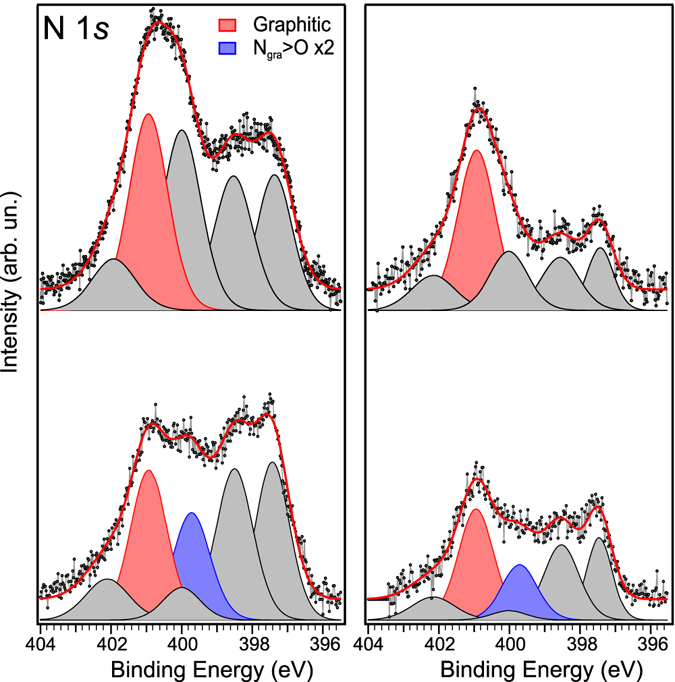
N 1 *s* core level spectra for the two N-graphene samples annealed to 300 (left) and 600 °C (right) before (top) and after (bottom) the exposure to molecular oxygen. Highlighted in colours are the graphitic components with (blue) and without (red) oxygen atoms bonded in epoxy configuration with the nearest carbon neighbours.

Analysis of the N 1 *s* core level spectra before and after the oxygen exposure, shown in Fig. 4, reveals the signature of oxygen dissociation. The nitrogen amount after interaction with molecular oxygen stays stable (within the estimated error of ±0.5 at.%) at 2.5 at.% for the sample previously annealed to 600 °C, whereas it slightly decreases from 5.7 to 5.1 at.% for the sample annealed at 300 °C. The lineshape of the N 1 *s* response also changes in important ways. In particular, we see a decrease of the graphitic-N signal at 400.9 eV and the pyrrolic at 400.0 eV, which is a clear indication that the oxygen atoms are adsorbed on graphene in a way that perturbs the chemical environment of those particular nitrogen sites. Furthermore, the absence of nitrogen oxide components, expected at binding energies higher than 402 eV^43, 44^, indicates that the O_2_ dissociation, although catalysed by nitrogen, does not lead to chemisorption of oxygen atoms directly with nitrogen. In the fit of the N 1 *s* spectra in Fig. 4, when maintaining the same BE position of all components before and after interaction with O_2_, a further component (blue, N6) is needed to properly fit the spectra after exposure to oxygen. This sixth component is found in both cases at −1.2 eV from the graphitic peak (red), in good agreement with the shift caused by the presence of two oxygen atoms surrounding a graphitic-N site (Table 2; Fig. 3e). In the two examined cases (300 and 600 °C), the amount of this component is respectively equal to 0.9 and 0.4 at.% (see Table 1). By comparing the amount of N6 + N4 after oxygen exposure with the graphitic nitrogen (N4) before the interaction, we have in particular for sample #1 an excess of about 0.4 at. %. This extra contribution could come from three-fold coordinated nitrogen in a Stone-Wales defect. Experimentally, the binding energy of this component is the same of many other defective configurations (pyrrolic, adatoms, cyanide…) and coinciding with N3, therefore it is not possible to univocally identify it by XPS. The extra amount of N6 is in fact more marked on the sample heated at lower temperature, where a lower amount of “pyrrolic” is desorbed or transformed. From a theoretical point of view, as we explained before, both graphitic N and N in SW defects decrease the energy barrier for oxygen dissociation, contrary to pyridinic^5^.

Since only epoxy oxygen is thought to take part in the oxygen dissociation mechanism, its concentration of 1.1 and 0.8 at.% in the two samples calculated from the fit of Fig. 2a is important to consider here. We can correlate the amount of epoxy oxygen on the sample both to the amount of graphitic N present before the oxygen interaction, and to the graphitic N that shifted because of this interaction after the exposure to the gas. This comparison shows that the epoxy oxygen is directly proportional to the amount of graphitic N initially present in the sample, by a factor of 0.7. On the other hand, considering only those graphitic N sites that change their chemical environment and shift towards lower BEs, we respectively find 1.3 and 2.0 oxygen atoms per shifted graphitic N in the two samples. Even though the error of these estimations could be on the order of 50%, these values are clearly well within the predicted range. If the dissociation of oxygen was due to the catalytic activity of the Ir substrate, then it should have some relation to the amount of pyridinic-N since this is the most accessible location for O_2_ to reach the metal. On the contrary, the observed dissociation rate is lower for higher pyridinic content.

After the oxygen interaction, additional annealing steps of the samples were performed to monitor the desorption and rearrangement of the oxygen and nitrogen species adsorbed on graphene. The results, presented in Supplementary Fig. S3 (O 1 *s*) and Supplementary Fig. S4 (N 1 *s*), show that the oxygen dissociation is an almost reversible process. The faster desorption of the oxygen component at 530.6 eV with respect to the one at 532.3 eV, which remains almost constant, is in agreement with thermal desorption observed on graphene oxide^45, 46^ and graphite^30^. These results further confirm our assignment to epoxy (shifted because of the proximity of graphitic nitrogen, see Table 2) and to ether. In particular, the remaining intensity at 415°C can be attributed to carbonyl, since at this temperature epoxy is not expected to be still present on graphene while C=O are stable^30, 33^.

Concerning the N 1 *s* evolution (after O_2_ interaction), there is a decrease of the N6 component, in agreement with the desorption of epoxy-oxygen and a partial restoring of the original graphitic-N. The decrease of the N1 component may be due to a reorganization of the graphene flakes after annealing, resulting in reduced contact with the underlying Ir. During this heating step the nitrogen amount further decreases from 5.1 to 4.6 at.%.

Since iridium itself is catalytically active, even though we have excluded oxygen intercalation and thus its obvious role in the dissociation, we repeated the experiment using *ex-situ* grown graphene on copper foil to compare with an inert substrate. In the case of graphene on copper, we also found an O uptake of ~2.0 at. % after exposure. The N 1 *s* core level spectra before and after O_2_ interaction are shown in Supplementary Fig. S5. The N 1 *s* lineshape in N-graphene/Cu differs from N-graphene/Ir(111) because of its different morphology (smaller graphene flakes, presence of bilayer regions and absence of moiré modulation). However, interaction with O_2_ shows the same behaviour as in Fig. 4: a decreasing of the high binding energy side of the spectra that can be explained with the appearance of a new component (N6) shifted by −1.25 eV from the graphitic peak (N4), in accordance with the theoretical calculation and with the case of graphene/Ir(111). This comparison is a confirmation that the observed mechanism is independent from the substrate where graphene is grown and it is only due to the nitrogen doping of graphene.

### Angle-resolved photoemission

**Figure 5:**
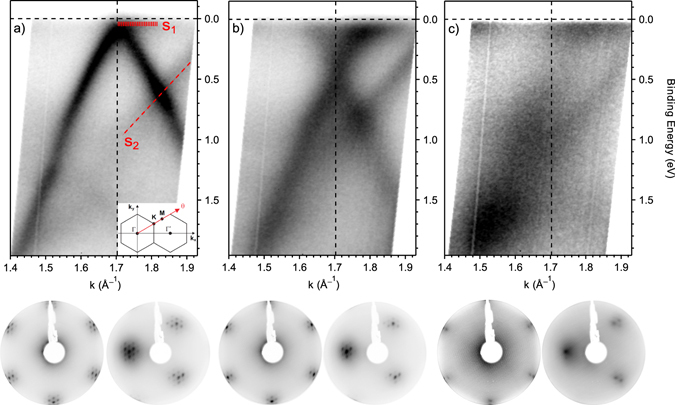
Top panels: band dispersion at the K point of the Brillouin zone obtained by plotting the ARPES intensity as a function of wave vector and binding energy for (**a**) pristine graphene/Ir(111), (**b**) after nitrogen doping and annealing at 600 °C and (**c**) after oxygen exposure. The red dashed lines indicate the two Ir surface states, S_1_ and S_2_. Inset: experimental geometry of the ARPES experiment. Bottom panels: the corresponding LEED patterns respectively recorded with an electron energy of 78 eV with the (0,0) spot at the centre of the image and at an off-angle of 10°.

The interaction of graphene with oxygen has important effects also on its valence band. Graphene on Ir(111) is an optimum sample since it grows in large domains in register with the hexagonal symmetry of the substrate and can be easily studied by angle-resolved photoemission spectroscopy (ARPES). We repeated the same procedure, with the same plasma source, at the BaDElPh beamline^47^ of the Elettra synchrotron (Trieste, Italy) on a freshly grown graphene/Ir(111) sample. According to the experimental geometry and the use of linearly polarized light, the anisotropy of the two branches of the Dirac cone is enhanced^48^. In Fig. 5 we therefore report the ARPES images obtained by the sum of the spectra in *p* and *s* polarization. In our analysis, we concentrated on the Dirac cone region along the Γ–K–M high symmetry direction of the Brillouin zone, as sketched in the inset of Fig. 5a. Pristine graphene, in Fig. 5a, shows the well-known linear dispersion of the π band of graphene (the Dirac cone), with the Dirac point’s location slightly above the Fermi level corresponding to a small amount of *p*-doping. In addition to the π band of graphene, we can also identify iridium surface states (indicated with S_1_ and S_2_) and minigaps (at 1.1 eV on the Γ–K branch and at 0.7 eV on the K–M), as already reported in literature^49^ and confirming the high quality of the present sample. The FWHM of the π band provides an indication of the disorder in the system. In particular, the FWHM of the MDC (moment distribution curves, a cut at constant energy of the ARPES spectrum) calculated at 0.3 eV BE for the pristine sample is 0.045 Å^−1^, slightly higher than the value (0.035 Å^−1^)^49^ reported for graphene grown on single-crystal Ir(111). This difference can be ascribed to the lower growth temperature used in the case of Ir(111) films on YSZ.

After nitrogen plasma exposure and subsequent annealing at 600 °C, a relatively large downshift of the Dirac cone takes place, with the Dirac point now located at 0.44 ± 0.02 eV and consistent with the significant *n*-doping action of graphitic nitrogen. The π band maintains its linear behaviour after doping and the surface states of iridium are still visible in the spectra of Fig. 5b, a confirmation of the integrity of graphene structure after plasma treatment. After doping, the FWHM of the π band MDC increases to 0.105 Å^−1^. This broadening is explained by the higher scattering rate induced by the nitrogen defects, but it is much lower than the one found in a similar experiment of graphene on copper^11^ (0.19 Å^−1^). This difference can be ascribed to the different morphology of the graphene layer caused by the two substrates: the moiré modulation present in graphene/Ir(111) and absent in graphene/Cu may play a role in hampering the graphene interaction with the metallic substrate. Indeed, the sharp long-range ordered super-symmetric over-layer due to the mismatch between graphene and Ir(111) is maintained after exposure to nitrogen plasma, as testified by the extra spots surrounding each diffraction spot in the hexagonal pattern of the low energy electron diffraction images (LEED) shown on the bottom panels of Fig. 5. The effect of oxygen exposure is visible in Fig. 5c: a broadening and a flattening of the π band occurs. This loss of order, which so strongly affects the band structure, is due to the attachment of oxygen atoms on the surface and the consequent change in the carbon hybridization from sp² to sp³. Also the LEED pattern testifies this modification with a strong increase in the background which almost hides the moiré superstructure. Only a few extra spots surrounding the two main ones are still visible, this is another confirmation of the absence of oxygen intercalated beneath the graphene. A bandgap of about 0.2 eV opens in the electronic spectrum at the K point; its centre can be evaluated at about 0.3 eV by the quenching of the intensity between the Fermi level and the Dirac point. This is similar to what has been observed for oxidized graphene/Ir(111)^50, 51^.

## Conclusions

We have studied the adsorption and dissociation of molecular oxygen on nitrogen-doped graphene directly with multiple *in situ* synchrotron spectroscopy experiments. The creation of single C-O bonds was highlighted by XPS, NEXAFS and ARPES measurements. Supported by first principles calculations, the modification of the N 1 *s* lineshape reveals a change in the chemical environment of graphitic nitrogen upon oxidation that is consistent with the adsorption of two oxygen atoms in epoxy position on the nearest carbon atom neighbours of the graphitic nitrogen, as predicted theoretically. A possible participation in this mechanism of three-fold coordinated nitrogen in a Stone-Wales defect can not be excluded. We ruled out any possible role played by the catalytically active iridium substrate in the oxygen dissociation by carefully comparing with previous studies on oxygen intercalated graphene/Ir(111). To further exclude its involvement in this mechanism, we compared our results with graphene on copper foil, a catalytically inert substrate, finding very similar results. Our work thus offers the first direct experimental description of the first step in the oxygen reduction reaction, providing detailed atomic-level understanding of the role of the nitrogen active sites in the interaction with molecular oxygen. Such knowledge will be crucial in understanding and improving the use of this novel metal-free material in gas-sensing devices and fuel cells.

## Methods

### Sample preparation

Graphene was grown *in situ* by the chemical vapour deposition of ethylene in ultra-high vacuum (UHV; base pressure better than 1 × 10^−10^ mbar) on iridium(111) films evaporated on top of an epitaxial yttria-stabilized zirconia (YSZ) film, following an established recipe that allows to achieve a long-range ordered layer^52^. Prior to graphene growth, the Ir(111) substrate was cleaned in UHV via cycles of Ar^+^ ion sputtering and annealing at 940 °C. The quality of the sample was confirmed by XPS, ARPES and LEED before each experimental step. Nitrogen was incorporated in graphene/Ir(111) by exposing the sample to the downstream of a µ-wave plasma source fed with N_2_ gas in the 10^−5^ mbar range. Two distinct samples with very similar initial nitrogen content were grown and then annealed at either 300 or 600 °C. Exposure to oxygen was performed in the same chamber, with a pressure of 5 × 10^−5^ mbar of pure molecular oxygen (O_2_) while keeping the sample at 200 °C for 30 min and then cooled down in O_2_ atmosphere until reaching T < 100 °C.

### Synchrotron spectroscopy

All measurements were performed at the Elettra synchrotron radiation facility in Trieste (Italy). XPS and NEXAFS were performed at the SuperESCA beamline, with photon energies of 200, 400, 500 and 650 eV used respectively for the Ir 4 *f*, C 1 *s*, N 1 *s* and O 1 *s* core levels. Photoelectrons were collected at normal emission by a Phoibos electron energy analyser equipped with a homemade delay line detection system. Photoemission intensities were normalized to the beam intensity and number of scans and the binding energy (BE) scale was calibrated using the Fermi edge of the Ir substrate. The error bar on the atomic concentration is on the order of ±0.5 at.%. NEXAFS measurements across the C *K*-edges were taken in Auger yield mode, with (horizontal) linearly polarized radiation and with the electric field vector parallel to the surface plane (incidence angle γ = 0°, transverse electric field) by appropriately rotating the sample. ARPES measurements were performed at the BaDElPh beamline^47^ with a photon energy of 34 eV for both *p* and *s* polarization to highlight both branches of the Dirac cone (K point at 1.7 Å^−1^) along the high-symmetry direction Γ–K–M of the Brillouin zone. The overall energy and angular resolution was set to 50 meV and 0.3°. At both beamlines, the full experiment (graphene growth, nitrogen doping and oxygen exposure) was performed *in situ* without breaking UHV conditions. XPS spectra were fitted using a commercial software (CasaXPS), with a Doniach-Sunjich line shape convoluted with a Gaussian used for the C 1 *s* (C-C sp²) and Ir 4 *f* core levels, while the other peaks had Voigtian line shapes. The FWHM of the N 1 *s* components reported in Table 1 is 1.2 eV for N1, 1.3 eV for N2, N3, N4, N6 1.4 eV for N5.

### Simulations

For atomistic simulations, we used density functional theory modelling implemented in the GPAW simulation package^53^. For each of the studied structures, we used a 6 × 6 supercell of graphene with 10 Å of vacuum, the Perdew-Burke-Erzerhof (PBE) functional, and a Monkhorst-Pack ***k***-point mesh of 5 × 5 × 1 (these parameters are sufficient for core level binding energies converged to within 100 meV)^42^. After introducing a N site, we relaxed the structure until maximum forces were <0.02 eV/Å. For calculations of the C, N and O 1 *s* core level binding energies, we utilized a standard delta Kohn–Sham (ΔKS) calculation with a projector-augmented wave dataset including an explicit core-hole on an atom of interest^40, 42, 54^.

Since the simulation method slightly underestimates the binding energies^42^, we corrected the calculated values by aligning the C 1 *s* energy of a C atom far away from the N site to the experimental C 1 *s* energy of 284.16 eV. However, since the error depends on the core level in question^40^, to facilitate direct comparison with the experiment, we also corrected the N 1 *s* to match the experimental graphitic N and the O 1 *s* to match the known value for epoxide on pristine graphene. Note that since this affects all 1 *s* levels of each element identically, the alignment does not affect the shifts upon oxidation or our conclusions.

## Electronic supplementary material

Supplementary Information
