# Supplementary material for: Spectroscopic observation of oxygen dissociation on nitrogen-doped graphene
Source: Sci Rep. 2017 Aug 11;7:7960. doi: 10.1038/s41598-017-08651-1 (PMC5554215; doi:10.1038/s41598-017-08651-1)
Supplement: Supplementary file 1 — Supplementary Information [file 41598_2017_8651_MOESM1_ESM.pdf]

SUPPLEMENTARY INFORMATION

Spectroscopic observation of oxygen dissociation on nitrogen-doped graphene

Mattia Scardamaglia[1,]* Toma Susi[2], Claudia Struzzi[1], Rony Snyders[1], Giovanni Di Santo[3], Luca Petaccia[3], Carla Bittencourt[1]

[1] Chemistry of Interaction Plasma Surface (ChIPS), University of Mons, Belgium

[2] University of Vienna, Faculty of Physics, Boltzmanngasse 5, A-1090 Vienna, Austria

[3] Elettra Sincrotrone Trieste, Strada Statale 14 km 163.5, 34149 Trieste, Italy

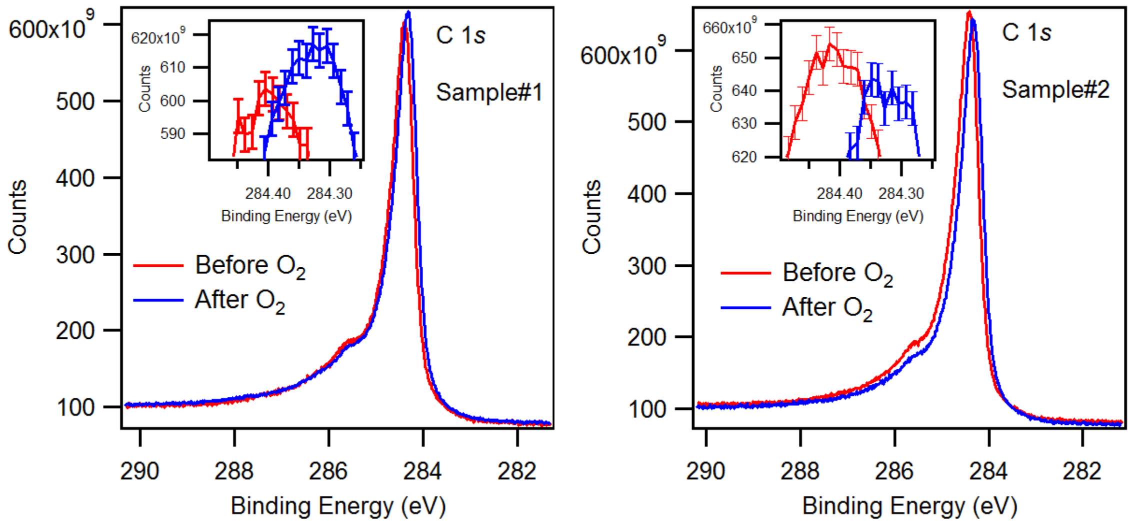

Figure S1. C 1*s* core level spectra before and after oxygen interaction for the two samples (#1: annealed after nitrogen plasma at 300 °C, #2: at 600 °C,).

The spectra are normalized to their relative beam current recorded in each file. In sample #1 (annealed to 300°C after nitrogen plasma), the intensity of the C 1*s* increases by 2.2 ± 0.8 %, this small variation could be due to the desorption of nitrogen still going on at this step. In the sample #2 (annealed to 600 °C after N plasma), the intensity of the C 1*s* decreases by 1.6 ± 0.8 %. The error bar has been estimated from the noise of the spectra. These results show that etching can be excluded; furthermore they corroborate our statement that as-doped N-graphene is not defective compared to partially covered iridium or defective graphene after cycles of intercalation/deintercalation [1,2]. Etching would also cause an increase in the C_{vac} peak in the C 1*s*, which is not the case here.

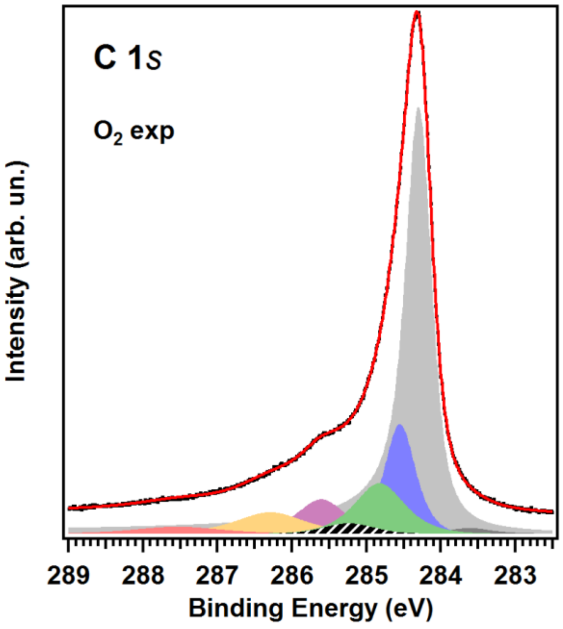

Figure S2. C 1*s* core level spectrum after oxygen interaction for sample#1. Black dots are the experimental data, the red continuous line is the fitting curve. The new component, related to C-O bond, is coloured in black and white stripes.

Due to the superposition of many different contributions in the 285-287 eV BE range, it is not straightforward to unequivocally assign all the peaks. In particular, since the oxygen uptake is low and, at the same time, there is a continuous evolution of the nitrogen species, the C 1*s* lineshape (Supplementary Figure S2), that is dominated by the sp^2 C-C contribution of graphene, does not show large variations (as in Figure 1c, before and after annealing). The FWHM of these peaks (from 0.5 eV to 1.3 eV) indicates that contributions from differently bonded carbon atoms may be present. We added another peak (black/white patterned) with free fitting parameters, while fixing the FWHM of the other peaks. This new component is found at 285.24 eV and accounts for 1.6 % of the area of the C 1*s*. This BE is consistent with a photoelectron emitted from a carbon atom participating in a C-O bond. The reorganization of the other peaks involves a decrease of all the C-N peaks, consistent with the lower amount of nitrogen, except for the two components at 284.82 eV (green) and 286.29 eV (yellow), previously assigned to contributions from sp^3 C-C and C-N bonds, respectively. The last one, in particular, is in good agreement with the values reported in Table 2 of the main article for C-O in proximity to a N atom, consistent with its unaltered intensity when compared to the other C-N components. The component at 284.82 eV, shifted by 0.52 eV from the main peak, is consistent with C atoms neighbouring epoxy groups, in agreement with Vinogradov et al. [3].

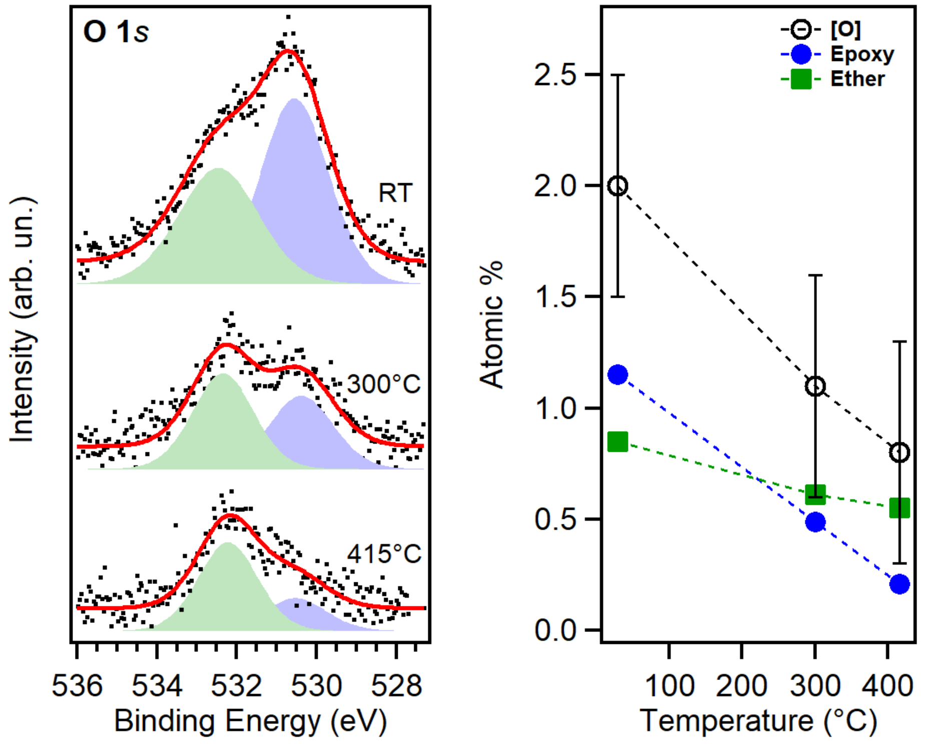

Figure S3. Evolution of the oxygen species after the oxygen interaction. a) From top to bottom, O 1s core level spectra after the oxygen interaction and subsequent annealing at 300 °C and 415 °C; b) the resulting oxygen content.

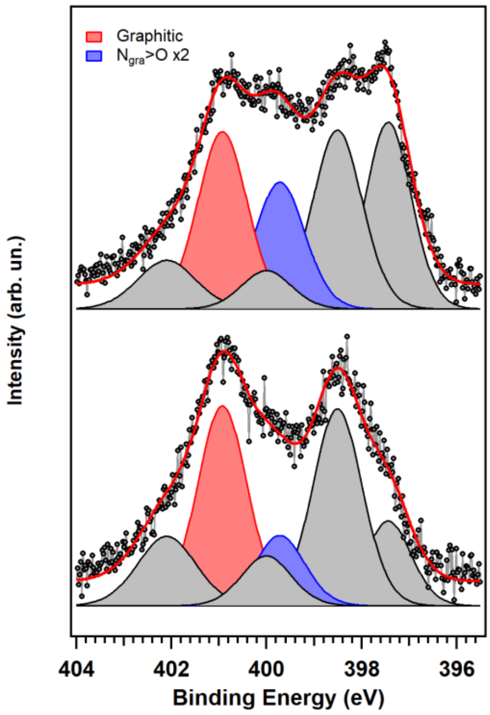

Figure S4. Evolution of the N 1*s* core level after the oxygen interaction (top) and subsequent annealing at 300 °C (bottom). Black dots are the experimental data, the red continuous line is the fitting curve. Highlighted in colours are the graphitic components with (blue) and without (red) oxygen atoms bonded in epoxy configuration with the nearest carbon neighbours.

After oxygen exposure, we studied the desorption and rearrangement of the oxygen and nitrogen species through further thermal annealing. The different thermal stability of the two O components (Figure S3) further supports their assignment to epoxy, with a faster desorption, and ether, mostly stable at higher temperature.

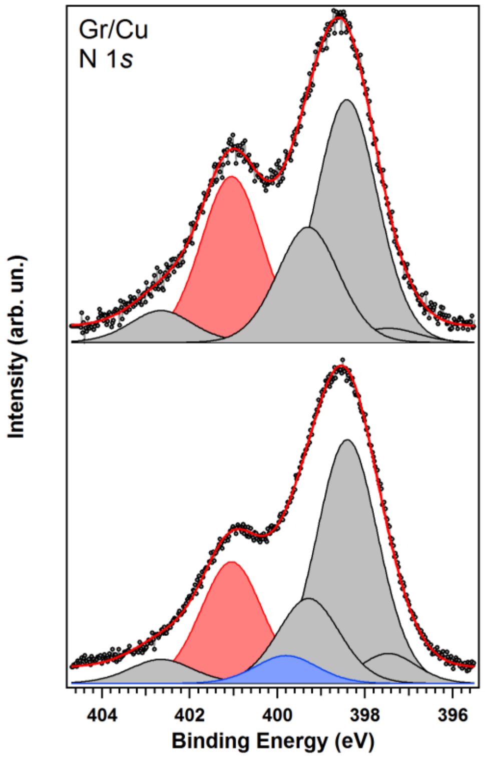

Figure S5. O_2 interaction with N-graphene/Cu foil. N 1*s* core level spectra on graphene/cu foil before (top) and after (bottom) the oxygen exposure. Black dots are the experimental data, the red continuous line is the fitting curve. Highlighted in colours are the graphitic components with (blue) and without (red) oxygen atoms bonded in epoxy configuration with the nearest carbon neighbours.

Since iridium itself is catalytically active, even though we have excluded oxygen intercalation and thus its obvious role in the dissociation, we repeated the experiment using *ex-situ* grown graphene on copper foil to compare with an inert substrate. The O_2 interaction mechanism and dissociation on N-graphene have a similar behaviour on both substrates, including the decrease of the high-BE component of the N 1*s* lineshape related to graphitic-N (see Figure S5). We prefer to focus on graphene on Ir, because in this case the whole process was performed *in situ* and it was oxygen-free. On the contrary, in the case of graphene on Cu foil, we have used an *ex-situ* grown graphene; an oxide layer is already present which decouples the graphene from the metal [4,5], preventing the evaluation of the oxygen components.

		Bulk	Surface	Surface2
Sample #1	BE (eV)	60.89	60.40	
gr/Ir(111)	FWHM (eV)	0.37	0.43	
	Rel. Area %	47.9	52.1	
	BE (eV)	60.88	60.40	60.72
N plasma	FWHM (eV)	0.36	0.44	0.41
	Rel. Area %	37.5	26.5	35.9
	BE (eV)	60.89	60.40	60.70
300°C	FWHM (eV)	0.37	0.43	0.42
	Rel. Area %	47.5	41.3	11.2
	BE (eV)	60.88	60.40	60.70
O2 exposure	FWHM (eV)	0.38	0.44	0.42
	Rel. Area %	47.8	33.7	18.5

		Bulk	Surface	Surface2
Sample #2	BE (eV)	60.88	60.40	
gr/Ir(111)	FWHM (eV)	0.37	0.44	
	Rel. Area %	47.2	52.8	
	BE (eV)	60.88	60.41	60.73
N plasma	FWHM (eV)	0.36	0.45	0.42
	Rel. Area %	36.2	29.2	34.6
	BE (eV)	60.89	60.40	60.68
600°C	FWHM (eV)	0.37	0.44	0.42
	Rel. Area %	46.1	46.2	7.7
	BE (eV)	60.89	60.41	60.69
O2 exposure	FWHM (eV)	0.37	0.45	0.42
	Rel. Area %	46.9	38.7	14.3

Table S1. Ir $4f_{7/2}$ fitting parameters.

References

1. Schröder, U. A. *et al.* Etching of graphene on Ir(111) with molecular oxygen. *Carbon* **96,** 320–331 (2016).
2. Larciprete, R. *et al.* Oxygen switching of the epitaxial graphene-metal interaction. *ACS Nano* **6,** 9551–8 (2012).
3. Vinogradov, N. a. *et al.* Impact of Atomic Oxygen on the Structure of Graphene Formed on Ir(111) and Pt(111). *J. Phys. Chem. C* **115,** 9568–9577 (2011).
4. Scardamaglia, M. *et al.* Tuning nitrogen species to control the charge carrier concentration in highly doped graphene. *2D Mater.* **3,** 11001 (2016).
5. Reckinger, N. *et al.* Oxidation-assisted graphene heteroepitaxy on copper foil. *Nanoscale* **8,** 18751–18759 (2016).
